# Associations between visceral adipose tissue estimates produced by near-infrared spectroscopy, mobile anthropometrics, and traditional body composition assessments and estimates derived from dual-energy X-ray absorptiometry

**DOI:** 10.1017/S0007114522003488

**Published:** 2023-08-14

**Authors:** Austin J. Graybeal, Caleb F. Brandner, Grant M. Tinsley, Hunter Haynes, Jon Stavres

**Affiliations:** 1 School of Kinesiology & Nutrition, College of Education and Human Sciences, University of Southern Mississippi, Hattiesburg, MS 39406, USA; 2 Department of Kinesiology & Sport Management, Texas Tech University, Lubbock, TX 79409, USA

**Keywords:** Digital anthropometry, Mobile health, Body composition assessment, Obesity

## Abstract

Assessments of visceral adipose tissue (VAT) are critical in preventing metabolic disorders; however, there are limited measurement methods that are accurate and accessible for VAT. The purpose of this cross-sectional study was to evaluate the association between VAT estimates from consumer-grade devices and traditional anthropometrics and VAT and subcutaneous adipose tissue (SAT) from dual-energy X-ray absorptiometry (DXA). Data were collected from 182 participants (female = 114; White = 127; Black/African-American (BAA) = 48) which included anthropometrics and indices of VAT produced by near-infrared reactance spectroscopy (NIRS), visual body composition (VBC) and multifrequency BIA (MFBIA). VAT and SAT were collected using DXA. Bivariate and partial correlations were calculated between DXA_VAT_ and DXA_SAT_ and other VAT estimates. All VAT indices had positive moderate–strong correlations with VAT (all *P* < 0·001) and SAT (all *P* < 0·001). Only waist:hip (*r* = 0·69), VAT_VBC_ (*r* = 0·84), and VAT_MFBIA_ (*r* = 0·86) had stronger associations with VAT than SAT (*P* < 0·001). Partial associations between VAT_VBC_ and VAT_MFBIA_ were only stronger for VAT than SAT in White participants (*r* = 0·67, *P* < 0·001) but not female, male, or BAA participants individually. Partial correlations for waist:hip were stronger for VAT than SAT, but only for male (*r* = 0·40, *P* < 0·010) or White participants (*r* = 0·48, *P* < 0·001). NIRS was amongst the weakest predictors of VAT which was highest in male participants (*r* = 0·39, *P* < 0·010) but non-existent in BAA participants (*r* = –0·02, *P* > 0·050) after adjusting for SAT. Both anthropometric and consumer-grade VAT indices are consistently better predictors of SAT than VAT. These data highlight the need for a standardised, but convenient, VAT estimation protocol that can account for the relationship between SAT and VAT that differs by sex/race.

Visceral obesity, defined as the excessive accumulation of adipose tissue around the organs within the abdominal cavity^(1)^, is a hallmark feature of the metabolic syndrome^(2)^. In particular, increased fat deposition in the visceral area is associated with an increase in insulin resistance, dyslipidemia, systemic inflammation, hypertension, and risk for type II diabetes and CVD^(3)^. In fact, visceral adipose tissue (VAT) accumulation is associated with the metabolic syndrome independent of obesity, where normal-weight individuals that present with a high degree of visceral adiposity often possess the aforementioned metabolic disorders^(4)^. As such, accurate and accessible assessments of VAT are critical in the detection and prevention of the metabolic syndrome and other metabolic abnormalities. However, there are several challenges in quantifying visceral adiposity that limit its application in routine care.

MRI and computed tomography (CT) are the most established VAT measurement techniques but are costly, inaccessible and generally unavailable for measurements of VAT outside of a research setting. Given the inherent obstacles associated with MRI and CT, simple waist circumference (WC) measures are commonly used as a proxy for visceral adiposity. However, WC measurements suffer from a high degree of technician dependency and an inability to distinguish VAT from its subcutaneous counterpart due to the superficial nature of the assessment^(5)^. Bioimpedance analysis (BIA) is a relatively available and easily operated method equipped to predict VAT; however, this method has shown mixed results for VAT estimations^(6,7)^,suggesting that some BIA VAT estimates are more associated with total body and subcutaneous adipose tissue (SAT) than VAT and requires rigid pre-assessment standardisation which limits its utility in practice. Dual-energy X-ray absorptiometry (DXA) scanning procedures have recently developed an imaging technique equipped to quantify VAT^(8)^. In fact, VAT estimates produced by DXA have been validated against CT in both adults^(9,10)^ and children^(8)^. However, the barriers associated with using DXA mirror those associated with MRI and CT. Because visceral obesity and traditional obesity progress concomitantly, and obesity continues to rise at an alarming rate, there is a vital need for accurate, cost-effective, and accessible assessment methods that can assess VAT remotely and during routine health evaluations.

The integration of mobile computing devices (i.e. smartphones) into healthcare systems has made a substantial impact on access to clinical care. What used to require high-powered computer systems or complex machinery can now, in part, be accomplished by a smartphone or a smartphone-operated healthcare tool that can fit in one’s pocket^(11,12)^. To that end, recent technological advancements have developed VAT estimation methods that can be conducted with, or operated by, a mobile application. Applications that conduct VAT estimates do so through visual body composition (VBC) estimates which collect two two-dimensional images to produce automated anthropometrics that predict VAT through artificial intelligence (AI) trained by clinical imaging procedures. Mobile healthcare tools also allow for near-infrared interactance (or near-infrared reactance spectroscopy, a.k.a. NIRS), a technique that emits near-infrared light capable of penetrating the abdominal area, to be operated by a smartphone and predict VAT from optical density readings. However, given their recent development and commercial introduction, these modalities have not been assessed against common clinical imaging techniques. Additionally, and as previously stated, most commercial devices are superficial in nature (compared with clinical imaging techniques) and demonstrate difficulty estimating deeper VAT apart from SAT due to the fact that large amounts of SAT are between placement of the device and the tissue it seeks to quantify (i.e. VAT) making it difficult to reach. As such, it is critical that a proposed VAT assessment method is equipped to delineate VAT from SAT given the varying health implications associated with each tissue and that excessive subcutaneous fat deposition precedes the spillover into visceral depots which may disrupt VAT estimation.

Given the rapid utilisation of mobile health applications for both patients and providers^(13)^, smartphone-based methods may offer a solution to the barriers associated with assessing VAT directly. Therefore, this study sought to evaluate the associations between VAT estimates produced by both consumer-grade devices (VBC, NIRS and BIA) and traditional anthropometrics and those derived from DXA before and after adjusting for SAT and if these indices are better predictors of VAT or SAT.

## Experimental methods

### Participants and ethical approval

A total of 187 individuals between ages 18 and 75 years were prospectively recruited for eligibility. Participants were excluded if they had a substantial amount of internal metal, were missing any limbs or part of a limb that would influence the accuracy of any device, had a pacemaker or any other electrical implant, and were pregnant, trying to become pregnant, breast-feeding, or lactating. This study was conducted according to the guidelines laid down in the Declaration of Helsinki, and all procedures involving human participants were approved by the University of Southern Mississippi ethics committee (IRB#21–213). Written informed consent was obtained from all participants.

### Procedures

Participants reported to the laboratory after an overnight fast from food, beverage, supplements and medication, and abstention from exercise for ≥ 8 h. Participants were then instructed to void their bladder and compared their urine to an eight-point colour chart to verify hydration (urine colour ≤ 6)^(14)^. Participants removed any remaining external metal and/or accessories and subsequently underwent several anthropometric assessments including height and weight collected by a digital stadiometer (SECA 769) and a calibrated digital scale (SECA), respectively. WC was collected by a flexible non-elastic aluminium tape measure at the level of the iliac crest^(15)^ and hip circumference at the largest lateral portion of the hips^(16)^. Waist and hip circumferences were conducted in duplicate by the same two investigators and averaged to produce a final estimate. Following anthropometric measurements, VAT was assessed by a portable NIRS device (Bello^®^, Olive Healthcare Inc.), multifrequency BIA (MFBIA; Tanita^®^ MC-780U), a VBC mobile application (myBVI^®^, Select Research LTD) and DXA (General Electric^®^). For VBC measures, participants were instructed to wear minimal form-fitting clothing which included only tights for males and tights/leggings and a sports bra for females. High-waisted shorts were adjusted to expose the participants belly button to the smartphone camera. Clothing alterations were made in the instance that any clothing was not form-fitting. Participants with long hair were instructed to tie their hair up so that no hair was below the shoulder area. Because smartphone applications are frequently updated to improve performance in practice, potential updates were checked daily and updated prior to testing when available. All application updates were defined as ‘performance improvements’ and ‘bug fixes’ unless stated otherwise.

### Visual body composition application

VAT estimates from VBC were collected using an iPhone 12 Pro^®^ (software version iOS 15·0·1, Apple^®^ Inc.). The VBC application software at the beginning and the end of the study were versions 3.0.0 and 3.1.1, respectively, and updates did not alter the internal algorithms. Each participant’s information was uploaded into the application prior to testing and included age, sex, height, and weight. All images were captured by the front-facing camera in front of a grey-coloured vinyl wall sticker so that only this area of the wall was with the scanning region. To avoid glare, opaque curtains were placed over all windows and images were collected in a specific area of the laboratory that did not have light at the participants back. The smartphone was attached to a tripod in a fixed location where the height of the tripod was positioned at an estimated average waist height (91·0 cm) and was the same for all participants. Once the smartphone was attached to the stationary tripod, the smartphone was fastened securely into place at an angle recommended by the application interface. For the assessment, participants stood so that mid-foot was 1·83 to 2·13 metres from the camera. Participants that were shorter were positioned closer to the camera to improve detection. Once positioned, participants were asked to stand in two separate poses. The first pose required the participant to stand in an ‘A-pose’ with arms and feet positioned away from the midline of the body. For the second image, participants turned to their left so that their right shoulder was facing the smartphone camera. Once participants were positioned, they were instructed to face forward and to fully extend their arms with their hands flat against their lateral thigh so that no part of the arm was outside the frame of their profile. All VBC assessments were conducted in duplicate to produce an index of VAT.

### Near-infrared reactance spectroscopy

VAT estimates were collected by a portable NIRS device operated by a smartphone application on the same iPhone^®^ as the VBC tests. The application software at the beginning and the end of the study were versions 2.0.2 and 3.0.2, respectively. A single update for this application included adding an additional language option. Prior to testing, the participant’s age, sex, height, weight and WC were uploaded into the application. The WC uploaded into the application was the average of the two tape measurements collected at the beginning of the procedures. After all information was uploaded, the device was synced with the mobile application to begin the assessment. Two scans were conducted for each measurement which required an investigator to place the device 2·54 cm (1-inch) above and below the belly button. To ensure accuracy, body hair in the scanning region deemed excessive by the investigators was shaven so that little to no hair impeded the device. On three occasions, hair within the scanning area could not be removed. For the measurement, an investigator placed the device in the designated regions using a light-emitting diode marker designed to show where the device should be placed in reference to the belly button. To prevent light pollution for the NIRS technology, the device was placed firmly against the abdominal area so that no other light impeded the signal. Measurements were conducted in duplicate, and a VAT index was produced.

### Multifrequency bioimpedance analysis

VAT index measured by bioimpedance was assessed using MFBIA with hand and foot electrodes. Because all prior measurements required the participant to stand, each participant stood for approximately 10 min before the MFBIA assessment. Participant information was uploaded into the device similar to the aforementioned methods. The MFBIA device used in this study required the selection of a ‘normal’ or ‘athletic’ setting, and the ‘normal’ setting was used for all participants.

### Dual-energy X-ray absorptiometry

DXA was conducted using the Lunar iDXA scanner with version 18 enCORE that includes CoreScan® for VAT and SAT estimates. As previously mentioned, DXA has shown to produce valid estimations of VAT^(8–10)^. Participants were positioned in accordance with the manufacturer guidelines. For larger participants who were unable to be completely scanned within the scanning dimensions, a reflection scanning technique was used so that the participants’ left side of the body was placed outside of the scanning dimensions allowing for a complete scan of the right side of the body which has shown to produce minimal error^(17)^. Whole-body percent fat (BF%) and android BF% were collected to describe the participants total abdominal adiposity. VAT and SAT area estimates from the android region were collected from the area between the iliac crest (inferior border of the measurement region) and 20 % of the distance between the iliac crest and the chin (superior border of the measurement region)^(10)^. VAT and SAT area (cm2), rather than volumes predicted from the area, were selected due to the two-dimensional nature of the DXA.

### Statistical analysis

Participant characteristics (Table 1) and descriptive measures of abdominal adiposity, including estimates from the devices in question (Table 2), are reported as mean ± 95 % CI. Three participants were excluded due to device errors which prevented VBC estimates. One participant was excluded for missing part of a limb that limited the ability to use MFBIA, and another was excluded due to size limitations that did not allow for appropriate reflection scanning and prevented accurate VAT and SAT estimation from DXA. Thus, 182 participants were included in the final analysis. A power analysis was conducted using a conservative correlation of *r* = 0·2 and determined that ninety-seven participants would be necessary to observe significant associations at an *α* = 0·05 between the devices/methods in question and DXA-derived values. All measurements were conducted in duplicate and averaged to produce a final estimate to be used in the final analyses. One participant had only one MFBIA and another had only one VBC assessment, and therefore, a single measurement was used as the final estimate for these participants. Multiple linear regression was used to model VAT estimates produced by DXA using traditional anthropometrics (height, weight, waist and hip circumference) and participant characteristics (sex, age, race and ethnicity). Following initial VAT modelling, VAT indices from each technical device (VBC, NIRS and MFBIA) were included into separate multiple regression models with the aforementioned anthropometrics and participant characteristics to determine if these devices offered additional contributions to VAT prediction. Pearson’s product-moment correlations and partial correlations were used to determine the association between VAT indices (VBC, NIRS, MFBIA, WC, waist:hip ratio, waist:height ratio and BMI) and VAT and SAT measurements produced by DXA (VAT_DXA_, SAT_DXA_). Partial correlations between VAT indices and VAT_DXA_ were adjusted for SAT_DXA_, and partial correlations between VAT indices and SAT_DXA_ were adjusted for VAT_DXA_. Separate traditional and partial correlations, as well as independent *t* tests for differences in abdominal adiposity using DXA, were conducted to examine associations by sex and race. For race, analyses were conducted for non-Hispanic White (F: 60·5 %; M: 39·5 %) and non-Hispanic Black/African-American (BAA) participants (F: 63·8 %; M: 36·2 %). Data from other racial and ethnic groups were included in the complete sample analyses only. Because precision metrics are important for the interpretation of the aforementioned results, but are outside the scope of the current analyses, precision analyses are presented in Supplementary Table 1. Statistical significance was accepted at *P* < 0·050. Data were analysed using IBM SPSS version 27 and Microsoft Excel version 16.

**Table 1.** Participant characteristics

	Total (*n* 182)		Female (*n* 114)		Male (*n* 68)	
Anthropometry	Mean	95 % CI	Mean	95 % CI	Mean	95 % CI
Age (years)	23·7	22·6, 24·7	23·1	21·9, 24·4	24·6	22·8, 26·5
Height (cm)	169·3	169·1, 170·6	164·9	163·7, 166·0	176·8	175·3, 178·3
Weight (kg)	77·3	74·4, 80·2	69·9	67·2, 72·7	89·6	84·7, 94·6
BMI (kg/m^2^)	26·8	26·0, 27·7	25·7	24·8, 26·7	28·8	27·1, 30·4
DXA BF (%)	30·3	28·9, 31·7	33·4	32·0, 34·9	25·0	22·6, 27·4
Race	*n*	%	*n*	%	*n*	%
White	127	69·8	78	68·4	49	72·1
Black/African-American	48	26·4	31	27·2	17	25·0
Asian	6	3·3	5	4·4	1	1·5
Native-American	1	0·5	0	0·0	1	1·5
Ethnicity						
Hispanic	14	7·7	10	8·8	4	5·9
BF classification						
≥ 40 %	36	19·8	28	24·6	8	11·8
30–39·9 %	57	31·3	46	40·4	11	16·2
20–29·9 %	60	33·0	35	30·7	25	36·8
< 20 %	29	15·9	5	4·4	24	35·3

DXA, dual-energy X-ray absorptiometry; BF, body fat.

Data presented as the mean and 95 % CI or as the *n* and % for each column total.

**Table 2.** Estimates of abdominal adiposity

	Total (*n* 182)		Female (*n* 114)		Male (*n* 68)		White (*n* 114)		Black/African-American (*n* 47)	
	Mean	95 % CI	Mean	95 % CI	Mean	95 % CI	Mean	95 % CI	Mean	95 % CI
Waist (cm)	89·5	87·4, 91·6	87·1*	84·7, 89·5	93·5	89·7, 97·4	87·7	85·4, 90·0	92·9	87·3, 98·2
Waist:hip	0·87	0·86, 0·88	0·86*	0·85, 0·87	0·90	0·88, 0·91	86·9	86·0, 87·9	88·0	85·9, 90·0
Waist:height	0·53	0·52, 0·54	0·53	0·51, 0·54	0·53	0·51, 0·55	0·52	0·50, 0·53	0·55†	0·52, 0·58
Android BF_DXA_ (%)	32·6	30·6, 34·6	34·1	31·8, 36·5	30·0	26·3, 33·7	30·5	28·0, 32·9	35·2	30·8, 39·6
DXA VAT_DXA_ (cm^2^)	55·5	46·2, 64·8	41·2*	33·5, 49·0	79·4	58·9, 99·9	51·1	39·3, 62·8	67·2	45·4, 89·1
DXA SAT_DXA_ (cm^2^)	170·2	150·8, 189·4	164·8	144·0, 185·7	179·0	140·3, 217·8	147·3	126·3, 168·4	213·4†	163·2, 263·6
NIRS Visceral Index	4·1	3·9, 4·3	3·9	3·7, 4·2	4·4	3·9, 4·8	4·0	3·8, 4·3	4·2	3·6, 4·8
VBC Visceral Index	2·3	2·1, 2·6	1·7*	1·5, 1·8	3·4	2·9, 3·9	2·2	1·9, 4·5	2·6	2·0, 3·3
MFBIA Visceral Index	4·4	3·7, 5·0	2·9*	2·5, 3·3	6·8	5·4, 8·3	3·8	3·1, 4·5	5·9†	4·1, 7·7

BF, body fat; DXA, dual-energy X-ray absorptiometry; VAT, visceral adipose tissue; SAT, subcutaneous adipose tissue; NIRS, near-infrared reactance spectroscopy; VBC, visual body composition; MFBIA, multifrequency bioimpedance analysis.

* Significantly different from male participants at *P* < 0·050.

† Significantly different from White participants at *P* < 0·050.

## Results

### Abdominal adiposity

All descriptive measures of abdominal adiposity were significantly different (all *P* < 0·050) between males and females other than android BF%_DXA_ (*P* = 0·06), waist:height ratio (*P* = 0·92), SAT_DXA_ (*P* = 0·52) and VAT_NIRS_ (*P* = 0·09) (Table 2). Only waist:height ratio (*P* = 0·04), SAT_DXA_ (*P* = 0·02) and VAT_MFBIA_ were significantly different between White and BAA participants. At each level of analysis, VAT_DXA_ had a significant and moderate-to-strong positive association with SAT_DXA_ (all *P* < 0·001; Table 3) which were highest in BAA participants (*r* = 0·89) and lowest for White participants (*r* = 0·74). Associations between VAT_DXA_ and SAT_DXA_ were similar between males (*r* = 0·81) and females (*r* = 0·83).

**Table 3.** Total and partial correlations between VAT indices and VAT and SAT estimates derived from DXA

	DXA visceral adipose area (cm^2^)§										DXA subcutaneous adipose area (cm^2^)||									
	Total (*n* 182)		Female (*n* 114)		Male (*n* 68)		White (*n* 114)		BAA (*n* 47)		Total (*n* 182)		Female (*n* 114)		Male (*n* 68)		White (*n* 114)		BAA (*n* 47)	
Waist (cm)	0·81‡	0·37‡	0·81‡	0·22*	0·84‡	0·37†	0·78‡	0·41‡	0·89‡	0·26	0·92‡	0·78 ‡	0·92‡	0·75‡	0·95‡	0·86‡	0·88‡	0·72‡	0·96‡	0·82‡
BMI (kg/m^2^)	0·80‡	0·33‡	0·79‡	0·15	0·80‡	0·24	0·76‡	0·36‡	0·87‡	0·22	0·90‡	0·73‡	0·92‡	0·76‡	0·92‡	0·77‡	0·86‡	0·68‡	0·94‡	0·72‡
Waist:hip	0·69‡	0·36‡	0·60‡	0·17	0·77‡	0·40†	0·69‡	0·48‡	0·75‡	0·17	0·68‡	0·28‡	0·63‡	0·30†	0·76‡	0·36†	0·58‡	0·14	0·78‡	0·40†
Waist:height	0·76‡	0·11	0·78‡	0·03	0·85‡	0·44‡	0·73‡	0·20*	0·85‡	–0·02	0·94‡	0·85‡	0·93‡	0·81‡	0·96‡	0·87‡	0·92‡	0·82‡	0·96‡	0·85‡
VAT_NIRS_	0·78‡	0·32‡	0·75‡	0·10	0·82‡	0·39†	0·75‡	0·36‡	0·84‡	–0·02	0·87‡	0·67‡	0·86‡	0·66‡	0·89‡	0·66‡	0·84‡	0·64‡	0·95‡	0·81‡
VAT_VBC_	0·84‡	0·60‡	0·79‡	0·38‡	0·86‡	0·50‡	0·84‡	0·67‡	0·85‡	0·26	0·77‡	0·33‡	0·80‡	0·43‡	0·93‡	0·76‡	0·70‡	0·21*	0·89‡	0·58‡
VAT_MFBIA_	0·86‡	0·61‡	0·80‡	0·31†	0·87‡	0·54‡	0·88‡	0·73‡	0·86‡	0·36*	0·81‡	0·42‡	0·86‡	0·58‡	0·93‡	0·77‡	0·74‡	0·26†	0·89‡	0·51‡
Unadjusted SAT (cm^2^)	0·79‡		0·83‡		0·81‡		0·74‡		0·89‡											

VAT, visceral adipose tissue; SAT, subcutaneous adipose tissue; DXA, dual-energy X-ray absorptiometry; BAA, Black/African-American; NIRS, near-infrared reactance spectroscopy; VBC, visual body composition; MFBIA, multifrequency bioimpedance analysis.

* Significant at *P* < 0·050.

† Significant at *P* < 0·010.

‡ Significant at *P* < 0·001.

§ Values are presented as: Pearson’s r (r_partial_ adjusted for SAT).

|| Values are presented as: Pearson’s r (r_partial_ adjusted for VAT).

### Visceral adipose tissue modelling

Results from the initial multiple linear regression model showed that age (*β* = 1·08, *P* = 0·003), height, weight, and waist and hip circumference (coefficients all *P* < 0·001) were all significant predictors of VAT. Sex, race and ethnicity were not significant predictors of VAT in this model (all coefficients *P* > 0·05). VAT_VBC_ (*β* = 17·77, *P* < 0·001) and VAT_MFBIA_ (*β* = 8·576, *P* < 0·003) were both significant contributors to the model after inclusion. In addition, age (*P* = 0·432) and weight (*P* = 0·061) were no longer significant predictors after including VAT_VBC_, whereas age, height and weight were no longer significant (all *P* > 0·05) after including VAT_MFBIA_. The inclusion of VAT_NIRS_ did not contribute to the VAT prediction model (*β* = 5·49, *P* = 0·243), and all significant predictors in the initial model remained significant.

### Associations between VAT indices and VAT_DXA_

**Fig. 1.**
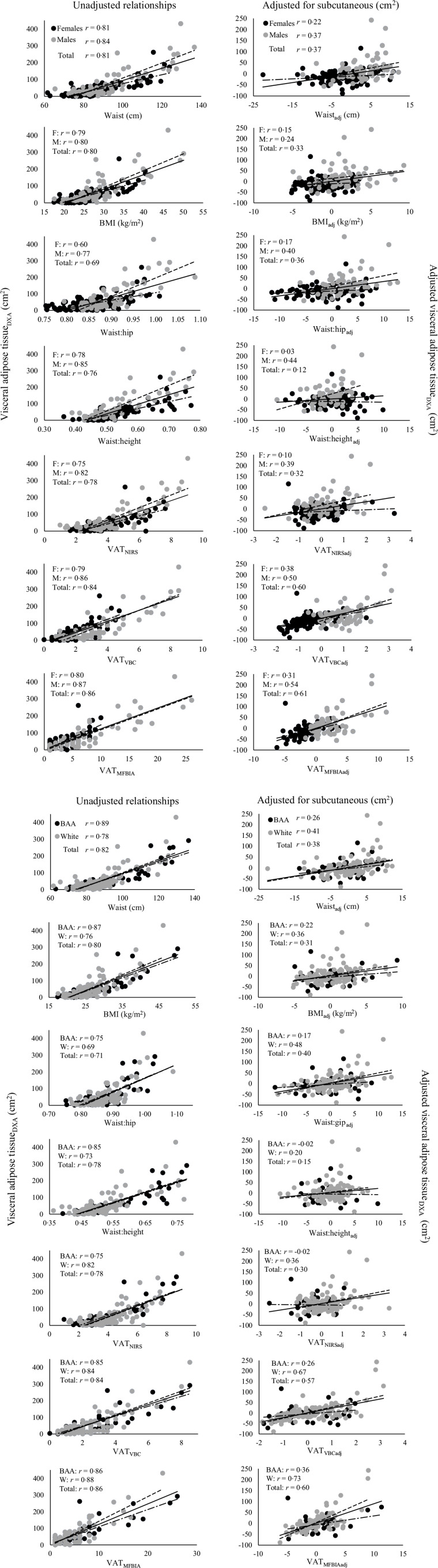
(a). Association between visceral adipose tissue indices and visceral adipose tissue estimates produced by DXA by sex. Scatterplots are displayed representing the relationship between each visceral adipose tissue index and visceral adipose tissue produced by DXA. Black markers represent female participants and grey markers represent male participants. The solid diagonal line represents the line of regression for the total sample, whereas the dashed line represents the relationship between males and the dashed-dotted line represents the relationship between females. The leftmost column displays the bivariate correlations for each group and the rightmost column displays the partial correlation for each group. (b) Association between visceral adipose tissue indices and visceral adipose tissue estimates produced by DXA by race. Scatterplots are displayed representing the relationship between each visceral adipose tissue index and visceral adipose tissue produced by DXA. Black markers represent Black/African-American participants and grey markers represent White participants. The solid diagonal line represents the line of regression for the total sample, whereas the dashed line represents the relationship between Black/African-American participants and the dashed-dotted line represents the relationship between White participants. The leftmost column displays the bivariate correlations for each group and the rightmost column displays the partial correlation for each group. DXA, dual-energy X-ray absorptiometry. BAA, Black/African-American; MFBIA, multifrequency BIA; NIRS, by near-infrared reactance spectroscopy; VAT, visceral adipose tissue; VBC, visual body composition.

Bivariate and partial associations between VAT indices and VAT_DXA_ and SAT_DXA_ are presented in Table 3. All complete and partial relationships by sex and the racial groups analysed in this study are illustrated in Fig. 1(a) and Fig. 1(b). For the total sample, all associations between VAT indices and VAT_DXA_ were significant moderate-to-strong and positive (*r* = 0·69 to 0·86, all *P* < 0·001) and were highest for VAT_MFBIA_ (*r* = 0·86) and VAT_VBC_ (*r* = 0·84). By sex, all associations between VAT indices and VAT_DXA_ remained significant moderate-to-strong and positive (all *P* < 0·001) with only waist:hip for females falling below the initial range observed for the total sample (*r* = 0·60). All VAT index Pearson’s r values differed by more than ± 0·05 between sex other than WC and BMI. Similar to sex comparisons, all associations remained significant moderate-to-strong and positive (all *P* < 0·001) for both White and BAA participants, and only WC (*r* = 0·89) and BMI (*r* = 0·87) for BAA participants fell above the range observed in the total sample. The only measurement methods that did not differ by Pearson’s r of more than ± 0·09 between White and BAA participants were waist:hip, VAT_MFBIA_ and VAT_VBC_.

### Associations between VAT indices and SAT_DXA_

For the total sample, associations between VAT indices and SAT_DXA_ were all significant moderate-to-strong and positive (*r* = 0·68 to 0·94, all *P* < 0·001; Table 3), and the only VAT indices that had a greater association with VAT_DXA_ than SAT_DXA_ were VAT_MFBIA_ and VAT_VBC_. The association between waist:hip and VAT_DXA_ was higher than the relationship between waist:hip and SAT_DXA_ but were nearly identical (*r* = 0·69 and *r* = 0·68, respectively). By sex, only waist:hip in males had a greater association with VAT_DXA_ than SAT_DXA_, but these associations were also nearly identical (*r* = 0·77 and *r* = 0·76, respectively). By race, only waist:hip, VAT_MFBIA_ and VAT_VBC_ had greater associations with VAT_DXA_ than SAT_DXA_ in White participants. All VAT indices had a greater association with SAT_DXA_ than VAT_DXA_ in BAA participants.

### Partial associations between VAT indices and VAT_DXA_ and SAT_DXA_

To further examine the influence that differing degrees of VAT and SAT adipose tissue had on the predictive ability of each VAT index, partial correlations were conducted controlling for the opposite adipose tissue (ex: associations with VAT_DXA_ controlling for SAT_DXA_). After controlling for SAT_DXA_ in the total sample, all associations between each VAT index and VAT_DXA_ remained significant except for waist:height (*P* = 0·15). However, associations between all VAT indices and VAT_DXA_ were reduced from moderate-to-large associations to small associations (*r* = 0·11 to 0·37) except for VAT_MFBIA_ and VAT_VBC_ which were reduced to from large associations to moderate associations (*r* = 0·60 and *r* = 0·61, respectively). SAT_DXA_ had the smallest influence on the associations between VAT_DXA_ and VAT_MFBIA_ and VAT_VBC_ (reduction in Pearson’s r of < 0·3). In the total sample, all associations been each VAT index and SAT_DXA_ remained significant after controlling for VAT_DXA_ and were robust to the influence of VAT_DXA_ (i.e. did not have a reduction in magnitude of association from moderate-to-large) except for waist:hip, VAT_MFBIA_ and VAT_VBC_ (Pearson’s r of < 0·45 after controlling for VAT_DXA_). Additionally, waist:hip, VAT_MFBIA_ and VAT_VBC_ were the only VAT indices with partial associations that remained higher for VAT_DXA_ than SAT_DXA_.

Males had a higher partial association for each VAT index and VAT_DXA_ after controlling for SAT_DXA_ than females across all methods. The only non-significant partial associations with VAT_DXA_ were for BMI in males (*r* = 0·24) and females (*r* = 0·15) and for waist:hip (*r* = 0·17) and waist:height (*r* = 0·03) in females only. However, the only partial association that was higher for the adjusted VAT_DXA_ than the adjusted SAT_DXA_ was waist:hip in males, but these associations were small and similar (*r* = 0·40 and *r* = 0·36, respectively). Similar findings were observed by race, where each VAT index and VAT_DXA_ association appeared to be more robust to the influence of SAT_DXA_ for White participants compared with BAA participants. Waist:hip, VAT_MFBIA_ and VAT_VBC_ in White participants were the only partial associations that were higher for the adjusted VAT_DXA_ than adjusted SAT_DXA_. In BAA participants, there were no VAT indices that had a greater partial association with the adjusted VAT_DXA_ than adjusted SAT_DXA_, and the only significant partial association with VAT_DXA_ was for MFBIA (*r* = 0·36). Interestingly, the only negative partial associations with VAT_DXA_ that occurred were observed for VAT_NIRS_ and waist:height in BAA participants. VAT_NIRS_ and waist:height in BAA participants were also amongst those most associated with SAT_DXA_ and appeared to be the most robust to adjusting for VAT_DXA_ when correlating with SAT_DXA_. Given that male participants were most resilient to the influence of SAT on VAT prediction while also having significantly more VAT than females, and that BAA participants were least resilient to the influence of SAT on VAT prediction while also having significantly more SAT, further analyses were conducted to determine the relationship between each VAT index and VAT_DXA_ and SAT_DXA_ across VAT and SAT quartiles by sex and race. The results of the full analysis by adipose tissue quartiles are presented in Supplementary Table 2(a), and the results of the subgroup analysis (sex and racial groups) by adipose tissue quartiles are presented in Supplementary Table 2(b). In summary, these data show that VAT indices have the greatest association with VAT at the highest level of VAT but also have the highest association with SAT at the highest levels of SAT. At the highest level of VAT, all VAT indices possessed a stronger correlation with VAT_DXA_ in males compared with females which appeared more resilient to the influence of SAT_DXA_. Interestingly, males in the highest quartile had more VAT_DXA_ on average (163·9 cm^2^, 95 % CI: 127·5, 200·4) compared with females (107·0 cm^2^, 95 % CI: 84·9, 129·1). By race and at the highest level of VAT, anthropometric VAT indices had stronger correlations with VAT_DXA_ in BAA participants, whereas the VAT devices appeared to have stronger associations for White participants. However, adjusting for SAT_DXA_ appeared to have a much greater influence on VAT index and VAT_DXA_ associations in both the third and fourth quartiles of VAT in BAA participants than in White participants. Additionally, BAA participants had more SAT_DXA_ on average in the third (178·2 cm^2^, 95 % CI: 144·8, 211·7) and fourth (412·4 cm^2^, 95 % CI: 355·4, 469·5) quartiles compared with White participants (171·2 cm^2^, 95 % CI: 163·2, 179·2; 340·9 cm^2^, 95 % CI: 288·0, 393·9, respectively).

## Discussion

The major findings of this study were that only waist:hip, VBC and MFBIA had stronger associations with VAT than SAT on a group level. However, our study showed that the higher partial associations between VBC, MFBIA and VAT occurred in White, but not male, female or BAA participants independently. Additionally, waist:hip was the only anthropometric method that had stronger bivariate and partial correlations with VAT but only when participants were male or White. We also showed that NIRS and waist:height had the weakest associations with VAT which were both highest in male participants but non-existent in BAA participants after adjusting for SAT. Overall, while these data demonstrate significant VAT prediction for certain methods during independent modelling not attributable to sex or race differences, these data show that the anthropometric and consumer-grade devices used in our study are better predictors of SAT than VAT, and that the predictive ability of each method varies by sex and race, likely due to the inherent differences in abdominal adiposity amongst these groups. While this study is unique in that it is the first to examine VAT estimation across multiple anthropometric and commercially available methods, it also highlights the need for a standardised, but convenient, estimation protocol equipped to overcome the relationship between subcutaneous and visceral adiposity which varies by race and sex.

Both MFBIA and VBC had the strongest correlations with VAT and were the only devices with both bivariate and partial correlations that were stronger for VAT than SAT. Several studies have reported significant correlations between BIA and VAT measured by CT^(7,18–21)^. Specifically, the MFBIA used in our study has demonstrated better predictive accuracy compared to single-frequency BIA^(22)^ which is likely because BIA devices that employ multiple channels are better equipped to measure abdominal impedance^(23)^. In fact, abdominal impedance alone has shown to produce a better index of VAT than whole-body MFBIA which indicates the importance of a standardised measurement site^(20)^. Conversely, other studies have reported that BIA is a better predictor of SAT than VAT^(6)^ or suggest that SAT has a significant influence on the ability of BIA to predict VAT^(20)^. Our findings show that MFBIA is relatively resilient to the influence of SAT on VAT prediction, but only for participants who are White. While it is possible that this due to racial/ethnic disparities that exist for BIA in BAA individuals^(24)^, it may be due to the fact that BAA participants in our study had significantly more SAT than White individuals. This is supported by previous studies showing that BAA individuals have inherently more SAT mass compared with White individuals^(25)^ and that SAT mass influences VAT estimation by MFBIA^(6,20)^. This may also be why MFBIA was a better predictor of SAT than VAT for male and female participants given the considerable proportion of BAA within each of these subgroups. While the weakened correlation between MFBIA and VAT may not necessarily be due to racial/ethnic biases in the measurement method, but rather the strong relationship between VAT and SAT given that large amounts of superficial SAT precede deposition into deeper VAT, it is difficult to recommend this VAT index for BAA individuals given the intrinsic differences in SAT mass and, thus, limits this method’s use in practice.

This is the only study, to our knowledge, to examine the utility of VBC as an index of visceral adiposity. While anthropometric measures have consistently shown to be better measures of SAT than VAT^(5)^ (as demonstrated in our study) and appear to be no better at predicting VAT than BMI^(26)^ (also observed in our study), anthropometrics generated from VBC use AI trained by clinical imaging techniques which should, theoretically, improve the predictive ability of this method and may explain why VBC had stronger correlations with VAT compared with more traditional methods. Additionally, VBC algorithms that rely on multiple body circumferences to predict VAT may have stronger prediction equations than WC or BMI alone. This may be why we observed a moderate correlation for waist:hip that was able to predict VAT to a slightly better degree than SAT^(27)^. Whole-body fat deposition patterns associated with visceral obesity may not be sufficiently described by WC, whereas waist:hip measurements demonstrate the degree of abdominal fat deposition relative to an area absent of visceral organs. As such, even at an above-average WC, a higher hip circumference would imply that fat deposition is more evenly distributed outside of the abdominal area and likely, a lower accumulation of VAT. However, both VBC and waist:hip were only better predictors of VAT after adjusting for SAT in particular instances. VBC, similar to MFBIA, was only a better predictor of VAT than SAT in White participants who, as previously mentioned, had significantly lower SAT than BAA participants. Because VBC is limited to measuring only the most superficial area of the abdomen, it is likely that VBC is unable to differentially estimate VAT at higher degrees of SAT given that a large amount of SAT likely occurs prior to excessive deposition in the visceral area. The weaker correlation for waist:hip after adjusting for SAT for all participants other than those who were either male or White may have occurred for multiple reasons. Because WC is a better predictor of SAT, an excessive WC without concurrent and similar increases in hip circumference may mathematically overpower the relationship between waist:hip and VAT. At a certain point of WC increases, waist:hip may become a better predictor of SAT due to the collinearity amongst SAT and VAT and the nature of the waist:hip equation. Additionally, the male abdominal region favours fat deposition compared with premenopausal females where males are more likely to accumulate fat in the visceral area (as our study demonstrated)^(28)^. Because males tend to deposit fat in the abdominal area, it is likely that VAT exponentially accumulates at higher levels of abdominal adiposity and may be why the only significant associations between waist:hip and VAT occurred at the highest levels of VAT in both the total sample (online Supplementary Table 2(a)) and in males (online Supplementary Table 2(b)).

NIRS operates by transmitting multiple wavelengths of light within the near-infrared band (750–2500 nm) into a tissue and recording the relative quantity of photons that are absorbed or scattered at these different wavelengths. Considering the known absorption coefficients of chromophores bound with oxygen (i.e. oxygenated Hb) at different wavelengths of NIR light, NIRS is able to provide relative indices of oxygenated Hb, deoxygenated Hb and total Hb within a tissue. This same logic is applied to the estimation of adipose tissue, where specific absorption coefficients across a range of NIR wavelengths can be used to estimate relative fat mass within the sampling volume. For that reason, time-resolved NIRS has potential value as a portable and easy-to-use tool for assessing visceral or subcutaneous adiposity. However, as is the case with any methodology, NIRS is subject to certain limitations. For instance, NIRS relies on the assessment of a superficial area of tissue, as opposed to DXA or MRI, which are able to assess entire cross sections of body segments. This means that the accuracy of NIRS-based assessments of adiposity are dependent on both the location of the measurement (hence, the reason for standardising the collection site) and the assumption of a strong relationship between visceral and subcutaneous adiposity. This may be especially true in cases where the NIRS signal may be saturated by the subcutaneous adipose layer. This is illustrated in our findings, where we report that the strength of association between VAT_NIRS_ and VAT_DXA_ decreases substantially when correcting for the relationship between visceral and SAT, especially in individuals within the highest quartile of subcutaneous adiposity (online Supplementary Table 2). We believe this to be the same reason for the lack of any significant relationship between VAT_NIRS_ and VAT_DXA_ within the lowest two quartiles of visceral adiposity. Specifically, the weak to non-significant associations between SAT_DXA_ and VAT_DXA_ within these two quartiles likely resulted in a failed estimation of visceral adiposity via NIRS. In contrast, the associations between VAT_NIRS_ and SAT_DXA_ were stronger across all groups and were more robust to corrections for the relationship between subcutaneous and VAT. Ultimately, these data support the notion that NIRS appears to be most effective at predicting SAT, and that the ability of NIRS to predict visceral adiposity is heavily dependent on the relationship between subcutaneous and visceral adiposity.

In conclusion, both anthropometric and consumer-grade VAT indices are more consistently better predictors of SAT than VAT. Although these approaches are user-friendly, easily operated and accessible, the superficial and non-invasive nature of each VAT measurement technique limits its ability to detect VAT independently of SAT which manifests in a lower predictive ability for groups with higher SAT mass such as BAA individuals. Moreover, the inherent differences in abdominal adiposity across sex and race limit the utility of each method and thus, they should be used with caution for clinical decision-making.

## Supplementary material

For supplementary material accompanying this paper visit https://doi.org/10.1017/S0007114522003488. click here to view supplementary material
