# Supplementary material for: Associations between visceral adipose tissue estimates produced by near-infrared spectroscopy, mobile anthropometrics, and traditional body composition assessments and estimates derived from dual-energy X-ray absorptiometry
Source: Br J Nutr. 2022 Oct 21;130(3):525–35. doi: 10.1017/S0007114522003488 (PMC10331432; doi:10.1017/S0007114522003488)
Supplement: Supplementary file 1 [file S0007114522003488sup001.docx]

**Supplementary Table 1.** Precision analysis of consumer-grade devices

|  | **Total**  **(n=182)** | **Female**  **(n=114)** | **Male**  **(n=68)** | **White**  **(n=114)** | **BAA**  **(n=47)** |
| --- | --- | --- | --- | --- | --- |
| VAT_NIRS_ | 0.952^‡^  (0.34) | 0.934^‡^  (0.34) | 0.966^‡^  (0.34) | 0.917^‡^  (0.42) | 0.999^‡^  (0.18) |
| VAT_VBC_ | 0.982^‡^  (0.22) | 0.937^‡^  (0.24) | 0.991^‡^  (0.19) | 0.972^‡^  (0.25) | 0.994^‡^  (0.17) |
| VAT_MFBIA_ | 0.998^‡^  (0.61) | 0.994^‡^  (0.18) | 0.999^‡^  (0.19) | 0.998^‡^  (0.19) | 0.999^‡^  (0.18) |

BAA, Black/African-American; VAT, visceral adipose tissue; NIRS, near-infrared reactance spectroscopy; VBC, visual body composition; MFBIA, multifrequency BIA. Data are presented as intraclass correlation coefficients using two-way random effects with absolute agreement and (precision error). ^‡^ significant at p < 0.001

**Supplementary Table 2a.** Associations Between VAT Indices and VAT/SAT Estimates From DXA by VAT and SAT Quartiles

|  | **DXA Visceral Adipose Area (cm^2^)^a^** | | | | | | | | |
| --- | --- | --- | --- | --- | --- | --- | --- | --- | --- |
|  | **Visceral Classification^b,c^** | | | |  | **Subcutaneous Classification^b,d^** | | | |
|  | **< 16.5**  **(n=45)** | **16.5-39.99**  **(n=47)** | **40.0-66.99**  **(n=44)** | **≥ 67.0**  **(n=46)** |  | **< 77.0**  **(n=46)** | **77.0-139.99**  **(n=47)** | **140.0-229.99**  **(n=44)** | **≥ 230.0**  **(n=45)** |
| Waist (cm) | 0.12  (0.03) | 0.21  (0.01) | 0.42^†^  (0.14) | 0.67^‡^  (0.46)^†^ |  | 0.16  (0.25) | 0.50^‡^  (0.43)^†^ | 0.41^†^  (0.37)^*^ | 0.89^‡^  (0.77)^‡^ |
| BMI (kg/m^2^) | 0.12  (0.06) | 0.23  (0.07) | 0.30  (-0.03) | 0.64^‡^  (0.39)^†^ |  | -0.06  (0.02) | 0.58^‡^  (0.53)^‡^ | 0.32^*^  (0.27) | 0.88^‡^  (0.77)^‡^ |
| Waist:hip | 0.03  (-0.03) | 0.00  (0.12) | 0.31^*^  (0.22) | 0.60^‡^  (0.37)^*^ |  | -0.19  (-0.12) | 0.10  (-0.01) | 0.01  (-0.09) | 0.61  (0.34)^*^ |
| Waist:height | 0.14  (0.04) | 0.23  (-0.03) | 0.46^†^  (0.20) | 0.56^‡^  (0.14) |  | 0.36^*^  (0.40)^†^ | 0.72^‡^  (0.69)^‡^ | 0.33^*^  (0.28) | 0.86^‡^  (0.78)^‡^ |
| VAT_NIRS_ | 0.03  (-0.06) | -0.00  (-0.30) | 0.34^*^  (-0.00) | 0.71^‡^  (0.53)^‡^ |  | 0.42^†^  (0.52)^‡^ | 0.26  (0.21) | 0.39^*^  (0.34)^*^ | 0.85^‡^  (0.69)^‡^ |
| VAT_VBC_ | -0.09  (-0.13) | -0.04  (-0.10) | 0.14  (0.10) | 0.76^‡^  (0.63)^‡^ |  | 0.01  (0.19) | 0.44^†^  (0.35)^*^ | 0.26  (0.17) | 0.81^‡^  (0.60)^‡^ |
| VAT_MFBIA_ | -0.03  (-0.06) | 0.01  (-0.14) | 0.39^*^  (0.26) | 0.77^‡^  (0.64)^‡^ |  | 0.14  (0.32)^*^ | 0.41^†^  (0.31)^*^ | 0.19  (0.07) | 0.80^‡^  (0.57)^‡^ |
| Unadjusted  SAT (cm^2^) | 0.15 | 0.37^*^ | 0.44^†^ | 0.56^‡^ |  |  |  |  |  |

^a^ Values presented as: Pearson r (r_partial_ adjusted); ^b^ adipose tissue area quartiles measured in cm^2^; ^c^ values are total and partial correlations between each VAT index and VAT_DXA_ after adjusting for SAT_DXA;_ ^d^ values are total and partial correlations between each VAT index and SAT_DXA_ after adjusting for VAT_DXA_ ^‡^ significant at p < 0.001; ^†^ significant at p < 0.010; ^*^  significant at p < 0.050.

**Supplementary Table 2b.** Associations Between VAT Indices and VAT Estimates From DXA by VAT Quartiles Across Race and Sex Groups

|  | **Visceral Classification (cm^2^)** | | | | | | | |
| --- | --- | --- | --- | --- | --- | --- | --- | --- |
|  | **< 16.5** | | **16.5-39.99** | | **40.0-66.99** | | **≥ 67.0** | |
|  | **W** | **BAA** | **W** | **BAA** | **W** | **BAA** | **W** | **BAA** |
|  | n=31  M=6 | n=11  M=0 | n=32  M=16 | n=12  M=6 | n=26  M=9 | n=10  M=3 | n=25  M=14 | n=14  M=8 |
| Waist (cm) | 0.17  (0.03) | 0.15  (0.23) | 0.07  (-0.19) | 0.54  (0.47) | 0.18  (0.01) | 0.73^*^  (0.23) | 0.64^†^  (0.44)^*^ | 0.83^‡^  (0.57)^*^ |
| BMI (kg/m^2^) | 0.13  (0.04) | 0.07  (0.08) | 0.11  (-0.03) | 0.56  (0.49) | -0.09  (-0.30) | 0.66^*^  (0.01) | 0.64^†^  (0.49)^*^ | 0.69^†^  (0.27) |
| Waist:hip | 0.13  (0.03) | -0.04  (-0.03) | -0.18  (-0.06) | 0.39  (0.47) | 0.21  (0.23) | 0.66^*^  (0.47) | 0.66^‡^  (0.51)^*^ | 0.47  (0.06) |
| Waist:height | 0.14  (-0.07) | 0.14  (0.19) | 0.21  (-0.05) | 0.34  (0.17) | 0.39^*^  (0.31) | 0.62  (-0.24) | 0.54^†^  (0.17) | 0.70^†^  (0.19) |
| VAT_NIRS_ | -0.03  (-0.13) | 0.03  (0.13) | 0.03  (-0.29) | -0.01  (-0.34) | 0.10  (-0.10) | 0.67^*^  (0.00) | 0.70^‡^  (0.56)^†^ | 0.68^†^  (0.09) |
| VAT_VBC_ | -0.15  (-0.18) | 0.03  (0.03) | -0.33  (-0.35) | 0.64^*^  (0.60) | -0.10  (-0.08) | 0.53  (0.44) | 0.84^‡^  (0.78)^‡^ | 0.61^*^  (-0.04) |
| VAT_MFBIA_ | -0.08  (-0.09) | 0.11  (0.23) | -0.35  (-0.50)^‡^ | 0.80^†^  (0.79)^†^ | 0.03  (-0.02) | 0.83^†^  (0.67)^*^ | 0.89^‡^  (0.84)^‡^ | 0.65^*^  (0.15) |
| Unadjusted  SAT (cm^2^) | 0.21 | -0.08 | 0.37^*^ | 0.30 | 0.26 | 0.73^*^ | 0.52^†^ | 0.74^†^ |
|  | **F** | **M** | **F** | **M** | **F** | **M** | **F** | **M** |
|  | n=39 | n=6 | n=23 | n=24 | n=31 | n=13 | n=21 | n=25 |
| Waist (cm) | 0.16  (-0.04) | -0.29  (0.58) | 0.39  (0.04) | 0.04  (-0.16) | 0.52^†^  (0.25) | 0.05  (-0.05) | 0.58^†^  (0.26) | 0.72^‡^  (0.64)^†^ |
| BMI (kg/m^2^) | 0.10  (-0.03) | -0.04  (0.31) | 0.30  (-0.07) | 0.19  (0.11) | 0.36^*^  (-0.11) | -0.01  (-0.12) | 0.56^†^  (0.20) | 0.67^‡^  (0.35) |
| Waist:hip | 0.06  (-0.05) | -0.54  (-0.29) | -0.01  (0.21) | 0.07  (0.08) | 0.46^*^  (0.39)^*^ | -0.22  (-0.28) | 0.44^*^  (0.03) | 0.62^†^  (0.38) |
| Waist:height | 0.24  (0.04) | -0.27  (0.22) | 0.25  (-0.08) | 0.21  (0.09) | 0.51^†^  (0.23) | 0.23  (0.18) | 0.47^*^  (-0.11) | 0.72^‡^  (0.58)^†^ |
| VAT_NIRS_ | 0.00  (-0.17) | 0.22  (0.59) | 0.33  (-0.02) | -0.21  (-0.38) | 0.48^†^  (0.19) | -0.18  (-0.37) | 0.44^*^  (0.06) | 0.79^‡^  (0.68)^‡^ |
| VAT_VBC_ | -0.19  (-0.25) | -0.25  (0.22) | -0.12  (-0.21) | 0.06  (-0.09) | 0.64^‡^  (0.53)^†^ | -0.40  (-0.64) | 0.59^†^  (0.33) | 0.76^‡^  (0.61)^†^ |
| VAT_MFBIA_ | -0.01  (-0.03) | -0.37  (0.00) | -0.04  (-0.21) | 0.05  (-0.13) | 0.62^‡^  (0.45)^*^ | 0.14  (0.07) | 0.52^†^  (0.20) | 0.77^‡^  (0.62)^†^ |
| Unadjusted  SAT (cm^2^) | 0.27 | -0.48 | 0.58^†^ | 0.19 | 0.48^†^ | 0.15 | 0.56^†^ | 0.61^†^ |

^a^ values are presented as: Pearson r (r_partial_ adjusted for SAT); ^b^ visceral adipose tissue area quartiles measured in cm^2^ ^‡^ significant at p < 0.001; ^†^ significant at p < 0.010; ^*^  significant at p < 0.050
